# Association Between Frailty and Inpatient Services Utilization Among Older Adults in Rural China: The Mediating Role of Multimorbidity

**DOI:** 10.3389/fmed.2022.818482

**Published:** 2022-02-01

**Authors:** Yemin Yuan, Jie Li, Peipei Fu, Chengchao Zhou, Shixue Li

**Affiliations:** ^1^Centre for Health Management and Policy Research, School of Public Health, Cheeloo College of Medicine, Shandong University, Jinan, China; ^2^Department of Epidemiology, School of Public Health, Cheeloo College of Medicine, Shandong University, Jinan, China; ^3^NHC Key Laboratory of Health Economics and Policy Research, Shandong University, Jinan, China

**Keywords:** frailty, multimorbidity, inpatient services utilization, older adults, rural

## Abstract

**Introduction:**

Developed and developing countries have different health systems and disease patterns. There is little evidence that frailty is related to inpatient services utilization in developing countries. In addition, the underlying mechanism of this relationship also remains unclear. This study aimed to examine the association between frailty and inpatient services utilization, and further explore whether multimorbidity play a mediating role in this association.

**Methods:**

A total of 3,242 rural older adults aged 60 and older were included in the analysis. Frailty was measured by the physical frailty phenotype (PFP). Multimorbidity and inpatient services utilization was measured based on participants' self-report and validated by village doctors. Ordered logistic regression analyses were performed to examine the association between frailty, multimorbidity and inpatient services utilization. Bootstrap analysis was further to explore the mediation effect of multimorbidity on frailty and inpatient services utilization.

**Results:**

The utilization of inpatient services was 20.1% (one: 15.8%, two or more: 4.3%). The prevalence of prefrailty and frailty was 64.7 and 18.1%, respectively. Frail older adults experienced a higher risk of multimorbidity and inpatient services utilization. Multimorbidity partially mediated the association between frailty and inpatient services utilization [95% confidence interval (CI): 0.005-0.016, *p* < 0.001]. The mediating effect of multimorbidity accounted for 19.0% of the total effect.

**Conclusions:**

Among Chinese rural older adults, frailty is associated with higher inpatient services utilization, and multimorbidity mediates this association. Recommendations are to increase frailty risk screening, chronic disease monitoring, and to do timely interventions.

## Introduction

China is the most populous country with the world's largest aging population. In 2018, about 250 million people were aged 60 years and above in China, and the number is expected to nearly double by 2050 (491 million) (1, 2). Such an enormous size of the aging population has put tremendous pressure on medical and health service supply in China. The Fifth National Health Service Survey in China found the utilization of inpatient services among older adults was higher than that of other age groups, and this utilization showed a rapid growth trend in the past decades (3). A recent study found that the inpatient services utilization among Chinese older people was higher in rural than in urban areas (4). The number of older people has increased dramatically. Therefore, to identify the influencing factors of inpatient services utilization among rural older adults is of great significance for the medical and health system.

Frailty is defined as a “clinically recognizable state of increased vulnerability resulting from aging-associated decline in reserve and function across multiple physiologic systems” (5). Frailty is strongly associated with a wide range of adverse health outcomes, such as falls, fractures, hospitalization, and death (6–8). Some studies examined the association between frailty and healthcare utilization using data from developed countries, such as Spain, Australia, and the United States, which indicated frailty was associated with higher hospitalization rates (9–12). There are different health systems in developed and developing countries (13). To date, it is largely unknown whether frailty is associated with inpatient services utilization among older adults in developing countries such as China. Previous studies found that the prevalence of frailty among Chinese older adults in rural areas was higher than that in urban areas (14, 15). However, there is no evidence that frailty is related to inpatient services utilization among Chinese rural older adults. In addition, the underlying mechanism of this relationship also remains unclear.

An increasing number of older people were found to be affected by more than one physical conditions (16). The co-occurrence of two or more physical chronic conditions in an individual was known as multimorbidity (17). Recently, more and more studies have focused on the association between frailty and multimorbidity. Studies found frailty was associated with multimorbidity among older adults, and frailty might predispose persons to the development of multiple chronic diseases (18, 19). A prospective cohort study in Italy found there was a significant association between frailty at baseline and incident multimorbidity in HIV outpatients (20). Furthermore, previous research revealed multimorbidity was associated with a reduction in life expectancy as well greater chances of hospitalization, poorer quality of life, and functional impairment (21). A prospective cohort study in the United States indicated that multimorbidity was independent predictors of higher inpatient utilization after considering conventional predictors (22). Disease patterns in developing countries differ from those in developed countries (23). There is evidence that 69.3% of older inpatients in China have multimorbidity (24). Thus, multimorbidity might be a mediator between frailty and inpatient services utilization.

In the current study, using the Shandong Rural Elderly Health Cohort (SREHC) baseline survey databases, we aim to 1) examine the association between frailty and inpatient services utilization, and 2) explore the mediating role of multimorbidity in the association between frailty and inpatient services utilization among older adults in rural China.

## Methods

### Data Source and Sample

Cross-sectional data were from the baseline survey of SREHC, which was conducted from May to June 2019 in Shandong province, China. Shandong is the second most populous province in China with 107 million people in 2018, with largest aging populations (1). We used a multistage stratified random sampling method to select the participants. More information about sample selection and data collection has been described in our previous publication (25). A total of 3,242 rural older adults with complete data were included in the analysis.

## Measures

### Inpatient Services Utilization

Inpatient services utilization was evaluated by the question that “Have you ever been hospitalized during the past 12 months?” Respondents with the answer of “yes” were further asked, “How many times have you been hospitalized?” The corresponding questionnaire covered main healthcare sectors of inpatient treatment in hospitals (including general hospital, specialized hospital, Chinese traditional medicine hospital, and township hospital), community healthcare center, and others. In this study, frequency of inpatient services utilization during the one year preceding the survey date was classified as zero, one or two or more times.

### Frailty

Frailty was measured by the physical frailty phenotype (PFP). The PFP included five criteria: shrinking, exhaustion, low physical activity level, slowness, and weakness (25, 26). The shrinking criterion was met if the respondent self-reported unintentional loss of at least 4.5 kilograms or 5% of body mass index (calculated from self-reported height and measured weight) in the past year. The exhaustion criterion was met if the participant answered “A moderate amount of time (3 to 4 days)” or “Most of the time (5 to 7 days)” when asked “How often during the last week did you feel this way?” to either of the two questions from the Center for Epidemiological Studies-Depression scale: “I felt everything I did was an effort” and “I could not get going”. The low physical activity was met if the total weekly of physical activity measured by International Physical Activity Questionnaire Short Form was <383 Kcal for men and <270 Kcal for women. The slowness criterion was met when gait speed, measured as the timed walk tests over a 4.6-metercourse, was at or below the gender- and height-specific cut-points. The weakness criterion was met when handgrip strength, assessed as the average of 3 readings by the dominant hand held dynamometer, was at or below the sex- and body mass index-specific cut-points. The respondents were scored 1 point for meeting one of the criteria. The total score ranges from 0 to 5 points. 0 point indicates nonfrail, 1-2 points indicates prefrail, and ≥3 points indicates frail.

### Multimorbidity

Multimorbidity was measured by the questions: “Have you ever been diagnosed with a chronic disease by a physician?”. If the answer was “yes,” the respondents would be further asked the questions that “How many chronic diseases have you ever been diagnosed?,” which was validated by the chronic disease case management system. In this study, multimorbidity referred to one individual with two or more chronic diseases. Participants were classified as having or not having multimorbidity. Participants with multimorbidity were further classified as having two chronic conditions or three or more. The list of diseases for the operationalization of chronic diseases was described in Supplementary File 1.

### Covariates

Demographic characteristics included age (continuous), gender (male, female), education (illiteracy, primary school, junior high school and above), marital status (married, unmarried/widowed/divorced), living arrangement (non-empty-nester, empty-nester), and household income [quintile 1 (the poorest), quintile 2, quintile 3, quintile 4 (the richest)]. Health status characteristics included physical exercise (no, yes), cognitive function (continuous), and self-reported health status (good, normal, bad). Physical exercise was measured by levels of frequency of exercise (27). Once a month or less is no physical exercise, more than once a month is physical exercise. Cognitive function was measured using the 30-item Chinese version of the Mini-Mental State Examination (MMSE) (28). The maximum score is 30 points, with higher scores indicating better cognitive function. MMSE was categorized further into mild/moderate/severe cognitive impairment (29).

### Statistical Analysis

We compared the characteristics of participants according to whether they use inpatient services, using Kruskal–Wallis test for continuous variables and Chi-square test for categorical variables. We examined the association among the main variables using Spearman's correlation analysis. The mediation test was based on the technique proposed by Wen and Ye (30). First, ordered logistic regression was employed to estimate the association between frailty and multimorbidity, and between frailty and inpatient services utilization, respectively. Second, ordered logistic regression was employed to further explore the association between frailty and inpatient services utilization when multimorbidity was included. We controlled covariates in above analyses. Finally, we performed bootstrap tests (sampling process was repeated 1,000 times) to examine the total, indirect and direct effect of the model (31). The indirect effect was regarded as statistically significant if the 95% confidence interval (CI) excluded zero. All tests were 2-sided with a significance level of *p* < 0.05. We conducted all analyses in Stata 14.2 (Stata Corp, College Station, TX).

## Results

### Sample Description

Table 1 shows the characteristics of the participants. Of the 3,242 respondents, 651 (20.1%) used inpatient services during the one year preceding the survey date. The utilization of inpatient services for prefrail and frail rural elderly was 18.9 and 29.5%, respectively. Approximately 64.7% of the respondents were prefrail, and 18.1% of those were frail. About one-third of respondents had two or more chronic diseases. Compared with respondents who did not use inpatient services, those who used inpatient services more likely to be older, be frail, and have multimorbidity. Diseases noted in groups with frailty and in groups with hospitalizations see Supplementary File 2.

**Table 1:** Characteristics of participants according to inpatient services utilization.

**Characteristics**	**Total,** ***N* (%)**	**Inpatient services utilization**			***p*-value**
		**Zero** **(*n* = 2,591)**	**One** **(*n* = 512)**	**Two or more** **(*n* = 139)**	
**Age (years)**, mean ± SD	70.1 ± 6.2	70.0 ± 6.1	70.7 ± 6.2	71.1 ± 6.7	0.007
**Gender**					0.094
Male	1,181 (36.4)	920 (35.5)	205 (40.0)	56 (40.3)	
Female	2,061 (63.6)	1,671 (64.5)	307 (60.0)	83 (59.7)	
**Education**					0.609
Illiteracy	1,353 (41.7)	1,093 (42.2)	204 (39.8)	56 (40.3)	
Primary school	1,257 (38.8)	1,006 (38.8)	200 (39.1)	51 (36.7)	
Junior high school and above	632 (19.5)	492 (19.0)	108 (21.1)	32 (23.0)	
**Marital status**					0.784
Married	2,415 (74.5)	1,937 (74.8)	376 (73.4)	102 (73.4)	
Unmarried/widowed/divorced	827 (25.5)	654 (25.2)	136 (26.6)	37 (26.6)	
**Living arrangement**					0.939
Non-empty-nester	590 (18.2)	469 (18.1)	96 (18.7)	25 (18.0)	
Empty-nester	2,652 (81.8)	2,122 (81.9)	416 (81.3)	114 (82.0)	
**Household income**					0.910
Quartile 1 (the poorest)	816 (25.1)	661 (25.5)	123 (24.0)	32 (23.0)	
Quartile 2	803 (24.8)	646 (24.9)	126 (24.6)	31 (22.3)	
Quartile 3	809 (25.0)	638 (24.6)	134 (26.2)	37 (26.6)	
Quartile 4 (the richest)	814 (25.1)	646 (25.0)	129 (25.2)	39 (28.1)	
**Physical exercise**					0.648
No	1,579 (48.7)	1,256 (48.5)	250 (48.8)	73 (52.5)	
Yes	1,663 (51.3)	1,335 (51.5)	262 (51.2)	66 (47.5)	
**MMSE (score)**, mean ± SD	22.9 ± 5.1	23.1 ± 5.0	22.6 ± 5.5	21.4 ± 5.1	<0.001
Mild cognitive impairment	1,263 (39.0)	1,004 (38.8)	195 (38.1)	64 (46.0)	
Moderate cognitive impairment	485 (15.0)	372 (14.4)	83 (16.2)	30 (21.6)	
Severe cognitive impairment	34 (1.0)	24 (0.9)	10 (2.0)	—	
**Self-reported health status**					<0.001
Good	1,456 (44.9)	1,292 (49.9)	136 (26.6)	28 (20.1)	
Normal	923 (28.5)	737 (28.4)	157 (30.6)	29 (20.9)	
Bad	863 (26.6)	562 (21.7)	219 (42.8)	82 (59.0)	
**Frailty status**					<0.001
Nonfrail	558 (17.2)	486 (18.7)	60 (11.7)	12 (8.7)	
Prefrail	2,097 (64.7)	1,701 (65.7)	317 (61.9)	79 (56.8)	
Frail	587 (18.1)	404 (15.6)	135 (26.4)	48 (34.5)	
**Multimorbidity**					<0.001
Zero or one chronic disease	2,101 (64.8)	1,792 (69.2)	252 (47.5)	57 (41.0)	
Two chronic diseases	801 (24.7)	578 (22.3)	170 (34.2)	53 (38.1)	
Three or more chronic diseases	340 (10.5)	221 (8.5)	90 (18.3)	29 (20.9)	

*SD, standard deviation; MMSE, Mini-Mental State Examination*.

### Correlation Analysis

Table 2 presents the correlation matrix of the association among frailty, multimorbidity and inpatient services utilization. Frailty was positively associated with inpatient services utilization (ρ = 0.139, *p* < 0.001). Multimorbidity was positively associated with frailty (ρ= 0.168, *p* < 0.001) and also positively associated with inpatient services utilization (ρ= 0.190, *p* < 0.001).

**Table 2:** Spearman's correlation analysis results of frailty, multimorbidity, and inpatient services utilization among older adults in rural China.

**Variable**	**1**	**2**	**3**
Frailty	1.000		
Multimorbidity	0.168***	1.000	
Inpatient services utilization	0.139***	0.190***	1.000

***p < 0.001.

### Mediating Effect of Analysis

Table 3 shows the relationship between frailty and multimorbidity among older adults in rural China. Frailty status were associated with multimorbidity. Prefrail (OR = 1.38, 95% CI: 1.10–1.74, *p* = 0.005) and frail (OR = 2.12, 95% CI: 1.60–2.81, *p* < 0.001) older people were more likely to have multimorbidity.

**Table 3:** The association between frailty and multimorbidity among older adults in rural China.

**Variable**	**OR**	**95% CI**	***p*-value**
**Frailty status**			
Nonfrail	1.00	1	
Prefrail	1.38	1.10–1.74	0.005
Frail	2.12	1.60–2.81	<0.001
**Age (years)**	0.99	0.98–1.01	0.465
**Gender**			
Male	1.00		
Female	1.46	1.23–1.72	<0.001
**Education**			
Illiteracy	1.00		
Primary school	1.08	0.90–1.30	0.416
Junior high school and above	0.94	0.74–1.20	0.645
**Marital status**			
Married	1.00		
Unmarried/widowed/divorced	1.06	0.88–1.28	0.541
**Living arrangement**			
Non-empty-nester	1.00		
Empty-nester	1.44	1.15–1.82	0.002
**Household income**			
Quartile 1 (the poorest)	1.00		
Quartile 2	1.00	0.80–1.24	0.978
Quartile 3	1.05	0.84–1.32	0.645
Quartile 4 (the richest)	1.12	0.87–1.43	0.375
**Physical exercise**			
No	1.00		
Yes	1.11	0.96–1.30	0.169
**MMSE (score)**	1.04	1.03–1.06	<0.001
**Self-reported health status**			
Good	1.00		
Normal	2.71	2.25–3.26	<0.001
Bad	5.04	4.15–6.11	<0.001

*OR, odds ratio; CI, confidence interval; MMSE, Mini-Mental State Examination*.

Table 4 shows the mediating role of multimorbidity on association between frailty and inpatient services utilization among older adults in rural China. The model without mediators (multimorbidity) showed that frailty was associated with inpatient services utilization (OR = 1.74, 95% CI: 1.24–2.44, *p* = 0.001). When the mediator was included, frailty was still associated with inpatient services utilization (OR = 1.59, 95% CI: 1.13–2.24, *p* = 0.008). Pre-frailty was not related to inpatient services utilization. Multimorbidity was also associated with inpatient services utilization. The more severe the multimorbidity, the more inpatient services may be used.

**Table 4:** The mediating effect of multimorbidity on association between frailty and inpatient services utilization among older adults in rural China.

**Variable**	**Model without mediators**			**Model with mediators**		
	**OR**	**95% CI**	***p*-value**	**OR**	**95% CI**	***p*-value**
**Frailty status**						
Nonfrail	1.00			1.00		
Prefrail	1.22	0.92–1.62	0.170	1.17	0.88–1.56	0.286
Frail	1.74	1.24–2.44	0.001	1.59	1.13–2.24	0.008
**Multimorbidity**						
Zero or one chronic disease				1.00		
Two chronic diseases				1.78	1.45–2.19	<0.001
Three or more chronic diseases				2.21	1.69–2.90	<0.001
**Age (years)**	1.02	1.00–1.03	0.040	1.02	1.00–1.03	0.029
**Gender**						
Male	1.00			1.00		
Female	0.74	0.61–0.91	0.003	0.70	0.58–0.86	<0.001
**Education**						
Illiteracy	1.00			1.00		
Primary school	1.06	0.85–1.33	0.598	1.06	0.84–1.33	0.617
Junior high school and above	1.35	1.01–1.80	0.041	1.37	1.03–1.84	0.032
**Marital status**						
Married	1.00			1.00		
Unmarried/widowed/divorced	1.11	0.89–1.39	0.363	1.10	0.88–1.38	0.401
**Living arrangement**						
Non-empty-nest elderly	1.00			1.00		
Empty-nest elderly	1.04	0.79–1.36	0.782	0.98	0.75–1.28	0.887
**Household income**						
Quartile 1 (the poorest)	1.00			1.00		
Quartile 2	1.24	0.95–1.61	0.116	1.23	0.94–1.61	0.123
Quartile 3	1.37	1.04–1.79	0.024	1.37	1.04–1.78	0.024
Quartile 4 (the richest)	1.50	1.11–2.02	0.008	1.48	1.10–2.00	0.010
**Physical exercise**						
No	1.00			1.00		
Yes	1.17	0.98–1.41	0.086	1.16	0.96–1.39	0.117
**MMSE (score)**	0.97	0.95–0.99	0.008	0.96	0.94–0.98	0.001
**Self-reported health status**						
Good	1.00			1.00		
Normal	1.91	1.51–2.40	<0.001	1.67	1.31–2.11	<0.001
Bad	3.91	3.11–4.92	<0.001	3.11	2.45–3.95	<0.001

*OR, odds ratio; CI, confidence interval; MMSE, Mini-Mental State Examination*.

**Figure 1:**
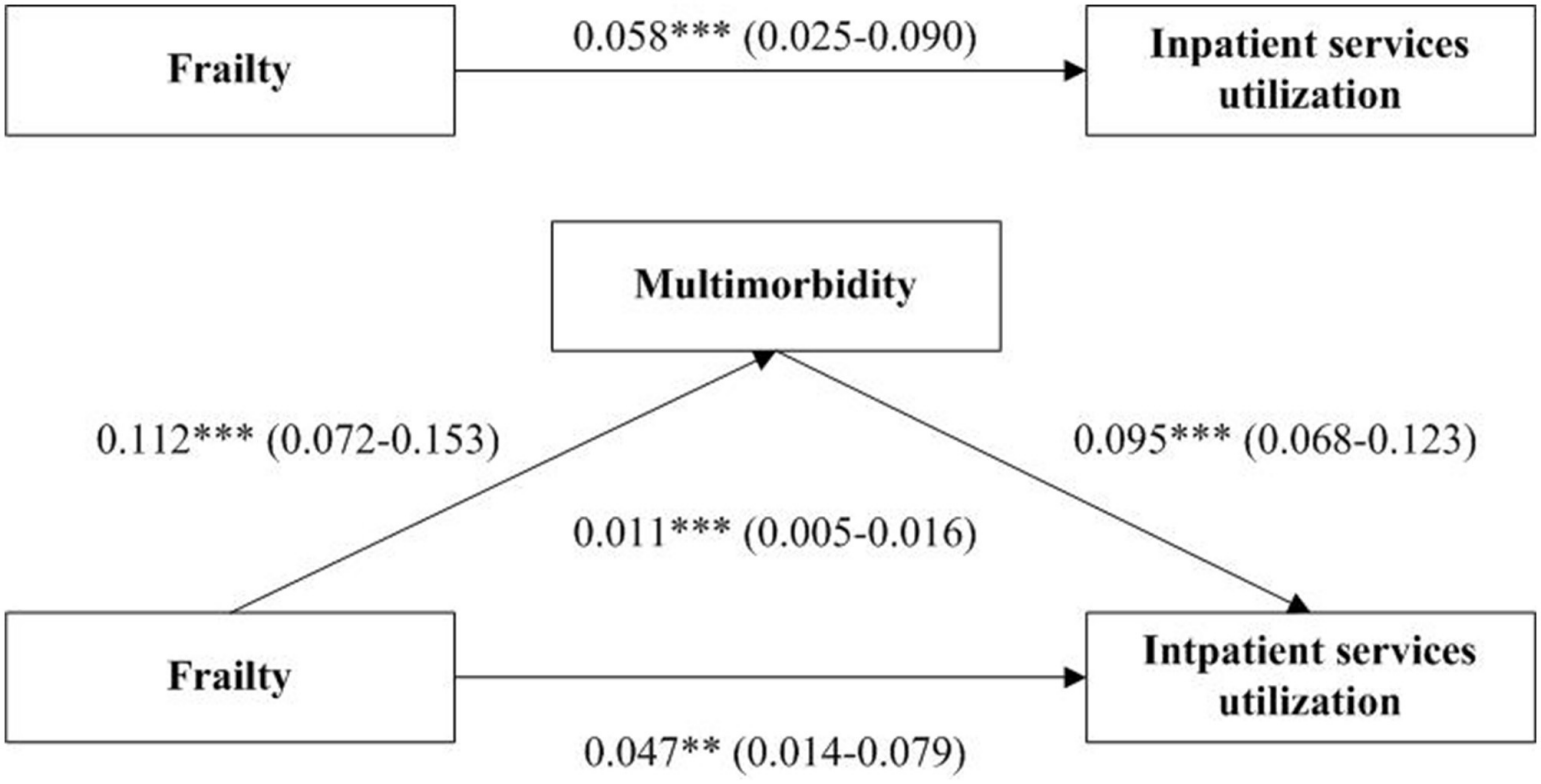
Path diagram of the association between frailty and inpatient services utilization in with multimorbidity as a mediator. **p < 0.01. ***p < 0.001. The coefficient and 95% confidence interval in the parentheses are shown. Models are adjusted for age, gender, education, marital status, living arrangement, household income, physical exercise, Mini-Mental State Examination score and self-reported health status.

Moreover, bootstrap test suggested that after adjusting for covariates, the total effect of frailty on inpatient services utilization was 0.058 (95% CI: 0.025–0.090, *p* < 0.001). The direct effect of frailty on inpatient services utilization was 0.047 (95% CI: 0.014–0.079, *p* = 0.005). The indirect mediating effect via multimorbidity was 0.011 (95% CI: 0.005–0.016, *p* < 0.001). These effects were significant since the 95% CI excluded zero. The association between frailty and inpatient services utilization was partially mediated by multimorbidity, of which, the indirect effect accounted for 19.0% of the total effect. Figure 1 illustrates the mediation pathway model with coefficients.

## Discussion

The current study found that frailty was associated with inpatient services utilization among older adults in rural China. Moreover, multimorbidity was associated with inpatient services utilization and partially mediated the association between frailty and inpatient services utilization.

This study found the utilization of inpatient services among Chinese rural older adults (60+) was 20.1%, higher than the national average (9.0%) of all age groups in rural areas (3), which indicated that older adults, as a special group, had a large demand for health services. The utilization of inpatient services in present study was higher than the 14.9% among older adults (60+) in rural Shandong province in 2013 (32). The finding suggests that the inpatient services utilization of older people is on the rise, which may be related to the deepening of medical reform and the basic coverage of medical insurance in recent years (33). Prefrail (18.9%) and frail (29.5%) rural elderly in Shangdong utilize more inpatient services than national average. In developed countries, the utilization of inpatient services for the prefrail elderly ranges from 24.2 to 50.2% (9–11, 34). The utilization of inpatient services for the frail elderly varies from 38.7 to 51.6% (9–11). These utilization rates are significantly higher than in our study. This could be attributed to better welfare in developed countries, since Chinese population would have to pay out of pocket.

Consistent with previous studies in developed countries, we found that frailty was positively associated with higher inpatient services utilization among rural older adults in China. A population-based study in Australia found that frailty was a risk factor for the use of inpatient services in the past year in older men, including spending at least one night in a hospital or nursing home (10). A prospective cohort study in the United States showed that frail older women had higher inpatient services utilization after accounting for multimorbidity and functional limitations (11). The association of frailty and inpatient services utilization may be related to the decline of various system functions among frail older adults (35). Frailty accelerates the process of functional decline and makes older adults more vulnerable to adverse health conditions. At the same time, the muscle strength of the frail elderly decreased and they were prone to fall, leading to fracture and head injury, etc. (36), which may increase the inpatient services utilization.

Inconsistent with prior studies in developed countries, we did not find a correlation between prefrailty and higher inpatient services utilization among older adults. A previous longitudinal study of older adults residing in Boston, United States found that prefrail participants were more likely to report hospitalization during the subsequent 10 months (37). Another longitudinal cohort study in Italy revealed that prefrail older adults account for the highest percentage of costs generated by using hospital services, as well as for the highest number of used hospital services (34). This difference in the association between prefrailty and inpatient services utilization may be attributed to the discrepancy between the level of economic and medical development across countries and regions. A formal welfare system for older adults is largely lacking in rural China. Moreover, there are a large number of low-income groups. For them, using inpatient services could consume most of their wealth, so they would not use the inpatient service at the stage of prefrailty. Perhaps when their physical conditions were getting worse, they and their family would choose to use inpatient services.

We also found that frailty was positively associated with multimorbidity, and frail older adults were more likely to have multiple chronic diseases. A recent systematic review and meta-analysis study found frailty was associated with an increasing risk of developing multimorbidity among older people (18). One possible explanation is the biological mechanism of the system failure process (35, 38). This frail system is more vulnerable to any stressor for older adults, hence increasing the risk of adverse health outcomes due to the inability to recover homeostasis. These adverse health outcomes would lead to an increased risk of multimorbidity. Multimorbidity was positively associated with inpatient services use, which was in consistent with previous studies (22). Some previous studies demonstrated that health care utilization and spending increased with the number of chronic conditions among older adults (39, 40). Agborsangaya et al. showed that persons with multimorbidity were twice as likely to be hospitalized or visit an emergency department compared to persons without multimorbidity (41). Since the aging process implies physiological decline, the probability of the occurrence of disease and functional disability increases with an increasing age. Multimorbidity may lead to more serious disability and deconditioning that have direct effects on health care utilization. From social aspects, the co-occurrence of chronic diseases requires regular medical appointments and special dietary needs. Due to a low income status, a lack of informal assistance and timely access to public and private healthcare systems, regular medical care and special dietary cannot be met. This means that multimorbid patient in rural areas do not having access to regular care, which increases inpatient utilization.

Furthermore, our results demonstrated multimorbidity played as a partial mediator between frailty and inpatient services utilization. Older adults with frailty were more likely to experience the co-occurrence of chronic diseases, which was related to a higher utilization of inpatient services. Although the prevalence of multimorbidity increases with an increasing age, it is not uncommon for an individual to experience multimorbidity before old age (42). Thus, regular surveillance for multimorbidity status (i.e., before a first chronic disease progresses to multimorbidity) could timely reduce adverse effects. Besides, multimorbidity was associated with a reduction in life expectancy as well a poorer quality of life and depression (21), which may increase the risk of hospitalization.

Our findings provided several implications. Recommendations are to increase risk screening, monitoring, and to do timely interventions. Firstly, frailty needs to be appropriately recognized in primary care rather than erroneously considered to be part of the normal aging process. General practitioner could lead risk screening initiatives to detect early frailty among older adults, thus enabling the health system to target these individuals more effectively. Secondly, better monitoring of multimorbidity and timely interventions might help to improve population health, to decrease inappropriate frequent access to more inpatient services. Moreover, to encourage general practitioner to follow them up regularly and meet their basic health service needs.

According to China's 7th national population census, 11 provinces have a total elderly population of more than 10 million, with Shandong being the only one with a population of more than 20 million. Perhaps our results have some reference significance for other provinces with large elderly population in China. In addition, the Chinese experience may also be beneficial to some less developed countries, as the living condition of rural China resembles that in other developing countries.

This study has several limitations. First, using self-reported data to measure variables may cause recall bias in some information. Second, due to the cross-sectional data, we could not determine the casual inference between frailty and inpatient services utilization. Future longitudinal studies are needed to elucidate the causal association. Finally, the proportion of mediating effect suggests that there may be other mechanisms, and future research can explore more factors which can explain other variance between frailty and inpatient services utilization.

## Conclusions

In conclusion, the utilization of inpatient services was 20.1% among Chinese rural older adults. Our findings revealed that frailty was associated with inpatient services utilization, and multimorbidity mediated this association. Recommendations are to increase frailty risk screening, chronic disease monitoring, and to do timely interventions.

## Data Availability Statement

The raw data supporting the conclusions of this article will be made available by the authors, without undue reservation.

## Ethics Statement

The protocol for this study was approved by the Ethical Committee of School of Public Health, Shandong University (approval number, 20181228). Participants were voluntary, and in review they had the right to withdraw from the study at any time. Written informed consent was obtained for all participants.

## Author Contributions

YY: conceptualization, methodology, formal analysis, writing-original draft, and writing-review and editing. JL: methodology and writing-review and editing. PF: investigation, resources, and data curation. CZ: conceptualization, supervision, writing-review and editing, and funding acquisition. SL: conceptualization, supervision, and writing-review and editing. All authors contributed to the article and approved the submitted version.

## Funding

This work was supported by the National Natural Science Foundation of China (Grant Numbers 71774104, 71473152, and 71974117), the China Medical Board (Grant Number 16-257), Cheeloo Youth Scholar Grant, and Shandong University (Grant Numbers IFYT1810 and 2012DX006).

## Conflict of Interest

The authors declare that the research was conducted in the absence of any commercial or financial relationships that could be construed as a potential conflict of interest.

## Publisher's Note

All claims expressed in this article are solely those of the authors and do not necessarily represent those of their affiliated organizations, or those of the publisher, the editors and the reviewers. Any product that may be evaluated in this article, or claim that may be made by its manufacturer, is not guaranteed or endorsed by the publisher.
