# Supplementary material for: Association Between Frailty and Inpatient Services Utilization Among Older Adults in Rural China: The Mediating Role of Multimorbidity
Source: Front Med (Lausanne). 2022 Feb 1;9:818482. doi: 10.3389/fmed.2022.818482 (PMC8844457; doi:10.3389/fmed.2022.818482)
Supplement: Supplementary file 1 [file Table_1.doc]

**Supplementary file 1: The list of diseases for the operationalization of chronic diseases**

**Chronic disease coding**

| **Coding** | **Disease Name** | **Coding** | **Disease Name** |
| --- | --- | --- | --- |
| **A. Nervous system** | | **038** | [Pancreatitis](javascript:;) |
| **001** | Alzheimer’s disease | **039** | Cholelithiasis |
| **002** | Other dementia | **040** | Cholecystitis |
| **003** | Schizophrenia | **041** | Cholangitis |
| **004** | Neurasthenia | **042** | Cystitis |
| **005** | Neurosis | **043** | Enterocele |
| **006** | Parkinson’s disease | **044** | Alcoholic cirrhosis |
| **007** | Cerebellar atrophy | **045** | Fatty liver |
| **008** | Trigeminal neuralgia | **046** | Liver cyst |
| **009** | Vertigo | **047** | Chronic hepatitis |
| **010** | Tinnitus | **048** | Ulcerative colitis |
| **011** | Epilepsy | **049** | [Peptic](javascript:;) [ulcer](javascript:;) |
| **012** | Cataract | **E.** [**Urogenital**](javascript:;) [**system**](javascript:;) | |
| **B.** [**Respiratory**](javascript:;) [**system**](javascript:;) | | **050** | Chronic renal failure |
| **013** | Chronic bronchitis | **051** | Chronic nephritis |
| **014** | Emphysema | **052** | Renal cyst |
| **015** | Chronic obstructive pulmonary disease | **053** | Nephrotic syndrome |
| **016** | Chronic pneumonia | **054** | Prostatic hyperplasia |
| **017** | Chronic rhinitis | **055** | Chronic pelvic inflammatory |
| **018** | Silicosis | **F. Endocrine, nutritional and metabolic** | |
| **019** | Asthma | **056** | Dyslipidemia |
| **C.** [**Circulatory**](javascript:;) [**system**](javascript:;) | | **057** | Diabetes |
| **020** | Hypertension | **058** | Gout |
| **021** | Atherosclerosis | **059** | [Obesity](javascript:;) |
| **022** | Coronary heart disease | **060** | [Nutritional](javascript:;) [deficiency](javascript:;) |
| **023** | Heart disease | **061** | [Vitamin](javascript:;) [deficiency](javascript:;) |
| **024** | Arrhythmia | **062** | Hyperthyroidism |
| **025** | Heart failure | **063** | Hypothyroidism |
| **026** | Insufficient blood supply to the heart | **G.** [**Musculoskeletal**](javascript:;) [**system**](javascript:;) | |
| **027** | Cor Pulmonale | **064** | Osteoarthropathy |
| **028** | Cerebrovascular disease | **065** | [Osteoporosis](javascript:;) |
| **029** | Aplastic anemia | **066** | Rheumatoid arthritis |
| **030** | Purpura | **067** | Rheumatoid arthritis |
| **031** | Low blood pressure | **068** | [Myasthenia](javascript:;) [gravis](javascript:;) |
| **D.** [**Digestive**](javascript:;) [**system**](javascript:;) | | **069** | Cervical and Lumbar Spondylopathy |
| **032** | Esophagitis | **H. Tumour** | |
| **033** | Chronic pharyngitis | **070** | Hemangioma |
| **034** | Chronic gastritis | **071** | lymphoma |
| **035** | Gastroptosis | **072** | [Liver](javascript:;) [cancer](javascript:;) |
| **036** | Chronic enteritis | **073** | [Gastric](javascript:;) [cancer](javascript:;) |
| **037** | Peptic gastric ulcer | **074** | Esophageal cancer |
| **Coding** | **Disease Name** | **Coding** | **Disease Name** |
| **075** | Lung cancer | **082** | Prostate cancer |
| **076** | Colon cancer | **083** | Tongue cancer |
| **077** | Rectal cancer | **084** | Leukemia |
| **078** | Breast cancer | **I.** [**Immune**](javascript:;) [**system**](javascript:;) | |
| **079** | [Pancreatic](javascript:;) [cancer](javascript:;) | **085** | [Systemic](javascript:;) [lupus](javascript:;) [erythematosus](javascript:;) |
| **080** | Bladder cancer | **100** | Other chronic diseases not mentioned above shall be assigned to 100 and the name of the disease shall be indicated |
| **081** | [Metrocarcinoma](javascript:;) |
