# Supplementary material for: Association Between Frailty and Inpatient Services Utilization Among Older Adults in Rural China: The Mediating Role of Multimorbidity
Source: Front Med (Lausanne). 2022 Feb 1;9:818482. doi: 10.3389/fmed.2022.818482 (PMC8844457; doi:10.3389/fmed.2022.818482)
Supplement: Supplementary file 2 [file Table_2.doc]

**Supplementary file 2: Diseases noted in groups with hospitalizations and in groups with frailty**

**Table S1** Diseases noted in groups with one inpatient service utilization.

| [**Name**](javascript:;) [**of**](javascript:;) [**disease**](javascript:;) | **Frequency** |
| --- | --- |
| **Hypertension** | **264** |
| **Coronary heart disease** | **142** |
| **Diabetes** | **87** |
| **Cerebrovascular disease** | **67** |
| **Dyslipidemia** | **34** |
| Cervical and Lumbar Spondylopathy | 34 |
| Chronic gastritis | 18 |
| Osteoarthropathy | 18 |
| Chronic bronchitis | 15 |
| Heart disease | 15 |
| Arrhythmia | 10 |
| Emphysema | 8 |
| Cataract | 7 |
| Rheumatoid arthritis | 6 |
| Asthma | 5 |
| Atherosclerosis | 5 |
| Rheumatoid arthritis | 5 |
| Cholelithiasis | 4 |
| Chronic hepatitis | 4 |
| Lung cancer | 4 |
| Neurosis | 3 |
| Hyperthyroidism | 3 |
| Cholecystitis | 3 |
| Ulcerative colitis | 3 |
| Gout | 3 |
| Heart failure | 2 |
| Esophageal cancer | 2 |
| Breast cancer | 2 |
| Chronic enteritis | 2 |
| Prostatic hyperplasia | 2 |
| Schizophrenia | 1 |
| Neurasthenia | 1 |
| Cerebellar atrophy | 1 |
| Trigeminal neuralgia | 1 |
| Vertigo | 1 |
| Tinnitus | 1 |
| Insufficient blood supply to the heart | 1 |
| Chronic pharyngitis | 1 |
| Hypothyroidism | 1 |
| Chronic obstructive pulmonary disease | 1 |
| Chronic pneumonia | 1 |
| Cor Pulmonale | 1 |
| Fatty liver | 1 |
| Cholangitis | 1 |
| Chronic renal failure | 1 |
| Hemangioma | 1 |
| Glaucoma | 1 |
| Purpura | 1 |
| Low blood pressure | 1 |

**Table S2** Diseases noted in groups with two or more inpatient service utilization.

| [**Name**](javascript:;) [**of**](javascript:;) [**disease**](javascript:;) | **Frequency** |
| --- | --- |
| **Hypertension** | **79** |
| **Coronary heart disease** | **51** |
| **Cerebrovascular disease** | **22** |
| **Diabetes** | **16** |
| **Cervical and Lumbar Spondylopathy** | **9** |
| **Chronic gastritis** | **9** |
| Dyslipidemia | 6 |
| Chronic bronchitis | 6 |
| Arrhythmia | 4 |
| Osteoarthropathy | 4 |
| Cataract | 3 |
| Asthma | 3 |
| Epilepsy | 2 |
| Emphysema | 2 |
| Lung cancer | 2 |
| Atherosclerosis | 2 |
| Rheumatoid arthritis | 2 |
| Cholelithiasis | 2 |
| Prostatic hyperplasia | 2 |
| Parkinson's disease | 1 |
| lymphoma | 1 |
| Heart disease | 1 |
| Breast cancer | 1 |
| Cor pulmonale | 1 |
| Cholecystitis | 1 |
| Alcoholic cirrhosis | 1 |
| Fatty liver disease | 1 |
| Rheumatoid arthritis | 1 |
| Rectal cancer | 1 |
| Colon cancer | 1 |
| Enterocele | 1 |
| Cystitis | 1 |
| Bladder cancer | 1 |

**Table S3** Diseases noted in groups with prefrailty.

| [**Name**](javascript:;) [**of**](javascript:;) [**disease**](javascript:;) | **Frequency** |
| --- | --- |
| **Hypertension** | **950** |
| **Coronary heart disease** | **354** |
| **Diabetes** | **249** |
| **Cervical and Lumbar Spondylopathy** | **149** |
| **Cerebrovascular disease** | **128** |
| Dyslipidemia | 95 |
| Chronic gastritis | 71 |
| Osteoarthropathy | 57 |
| Chronic bronchitis | 46 |
| Arrhythmia | 41 |
| Rheumatoid arthritis | 36 |
| Heart disease | 23 |
| Emphysema | 18 |
| Cataract | 18 |
| Asthma | 15 |
| Atherosclerosis | 12 |
| Rheumatoid arthritis | 12 |
| Cholelithiasis | 12 |
| Prostatic hyperplasia | 11 |
| cholecystitis | 10 |
| Ulcerative colitis | 9 |
| Hyperthyroidism | 8 |
| Insufficient blood supply to the heart | 7 |
| Fatty liver | 7 |
| Neurosis | 6 |
| Vertigo | 6 |
| Chronic pharyngitis | 5 |
| Parkinson's disease | 4 |
| Cor Pulmonale | 4 |
| Esophageal cancer | 4 |
| Low blood pressure | 4 |
| Neurasthenia | 3 |
| Lung cancer | 3 |
| Chronic enteritis | 3 |
| Chronic hepatitis | 3 |
| Chronic renal failure | 3 |
| Gout | 3 |
| Vitiligo | 3 |
| Glaucoma | 2 |
| Tinnitus | 2 |
| Chronic rhinitis | 2 |
| Heart failure | 2 |
| Chronic obstructive pulmonary disease | 2 |
| Hypothyroidism | 2 |
| Breast cancer | 2 |
| Gastric cancer | 2 |
| Trigeminal neuralgia | 1 |
| Cerebellar atrophy | 1 |
| Silicosis | 1 |
| Esophagitis | 1 |
| Gastroptosis | 1 |
| Peptic gastric ulcer | 1 |
| Alcoholic cirrhosis | 1 |
| Liver cyst | 1 |
| Cholangitis | 1 |
| Chronic nephritis | 1 |
| Renal cyst | 1 |
| Colon cancer | 1 |
| Rectal cancer | 1 |
| Cystitis | 1 |
| Bladder cancer | 1 |
| Varicose veins | 1 |
| Aplastic anemia | 1 |
| Hemangioma | 1 |
| Epilepsy | 1 |
| Purpura | 1 |

**Table S4** Diseases noted in groups with frailty.

| [**Name**](javascript:;) [**of**](javascript:;) [**disease**](javascript:;) | **Frequency** |
| --- | --- |
| **Hypertension** | **336** |
| **Coronary heart disease** | **160** |
| **Diabetes** | **89** |
| **Cerebrovascular disease** | **62** |
| **Cervical and Lumbar Spondylopathy** | **51** |
| Dyslipidemia | 27 |
| Chronic gastritis | 23 |
| Chronic bronchitis | 19 |
| Osteoarthropathy | 18 |
| Rheumatoid arthritis | 16 |
| Heart disease | 15 |
| Arrhythmia | 9 |
| Rheumatoid arthritis | 5 |
| Cholecystitis | 4 |
| Neurosis | 3 |
| Parkinson's disease | 3 |
| Cataract | 3 |
| Emphysema | 3 |
| Atherosclerosis | 3 |
| Aplastic anemia | 3 |
| Chronic hepatitis | 3 |
| Prostatic hyperplasia | 3 |
| Breast cancer | 3 |
| Lung cancer | 3 |
| Epilepsy | 2 |
| Asthma | 2 |
| Vertigo | 2 |
| Heart failure | 2 |
| Cor Pulmonale | 2 |
| Fatty liver | 2 |
| Gout | 2 |
| Low blood pressure | 2 |
| Schizophrenia | 1 |
| Neurasthenia | 1 |
| Trigeminal neuralgia | 1 |
| Insufficient blood supply to the heart | 1 |
| Cerebellar atrophy | 1 |
| Chronic pneumonia | 1 |
| Ulcerative colitis | 1 |
| Chronic nephritis | 1 |
| Hyperthyroidism | 1 |
| Hypothyroidism | 1 |
| [Osteoporosis](../../../../D:/Program%20Files%20(x86)/Dict/8.10.3.0/resultui/html/index.html#\\javascript:;) | 1 |
| Hemangioma | 1 |
| Enterocele | 1 |
